# Diabetic Mastopathy in a Patient with High Risk of Breast Carcinoma: A Management Dilemma

**DOI:** 10.7759/cureus.7003

**Published:** 2020-02-15

**Authors:** Salsabeel S Alkhudairi, Mohammed M Abdullah, Ahmed G Alselais

**Affiliations:** 1 Surgery Department, Imam Abdulrahman Bin Faisal University, Dammam, SAU; 2 General Surgery Department, King Fahad Hospital, Hufof, SAU; 3 Surgery Department, King Hamad University Hospital, Manama, BHR

**Keywords:** diabetic mastopathy, diabetes mellitus type i, breast carcinoma, lymphocytic mastitis, fibrous mastopathy, benign breast condition

## Abstract

Diabetic mastopathy is a rare benign breast condition. It is strongly associated with type I diabetes mellitus and commonly presents similar to malignancy. Here, we report a case of a 29-year-old Saudi female with a long history of type I diabetes mellitus (DM) who presented with a painless hard breast mass and had a strong family history of breast cancer. Further evaluation with ultrasound (US) imaging revealed a highly suspicious, ill-defined hypoechoic lesion. Mammographic examination revealed that both breasts were of normal shape with bilateral dense glandular parenchyma. US-guided true-cut biopsy was carried out, which showed acellular fibro-sclerotic tissues with normal-looking lobules and ducts surrounding by a dense lymphocytic infiltrate. Subsequently, a diagnosis of diabetic mastopathy was established. Results were discussed with the patient, and an agreement was reached to proceed with an excisional biopsy for further reassurance and exclusion of malignancy. Local surgical excision of the lesion was performed and histopathological examination revealed extensive fibrosis of the specimen with no cellular atypia. Awareness of such a condition, with its clinical, radiographical, and histopathological characteristics, is essential in order to alleviate the patient's anxiety and avoid unnecessary surgical interventions.

## Introduction

Diabetic mastopathy (DMP), previously known as "fibrous disease of the breast" is an uncommon, benign, fibro-inflammatory breast disease [1-3]. It classically affects women with type I diabetes mellitus (DM I) in the premenopausal period and especially those with other diabetic microvascular complications [2-3]. Although both clinically and radiographically, DMP presentation is commonly similar to a malignant process, it is neither a premalignant nor a malignant condition [2-4]. Soler and Khardori first described DMP in 1984 in 12 women with longstanding DM I and with similar breast symptoms [1]. Knowledge of DMP has considerably increased in recent years [2-3]. However, deficiencies remain in understanding its pathogenesis, and DMP management is controversial. Moreover, awareness regarding this condition is important, especially since Saudi Arabia is an area with a high prevalence of DM [5].

## Case presentation

**Figure 1:**
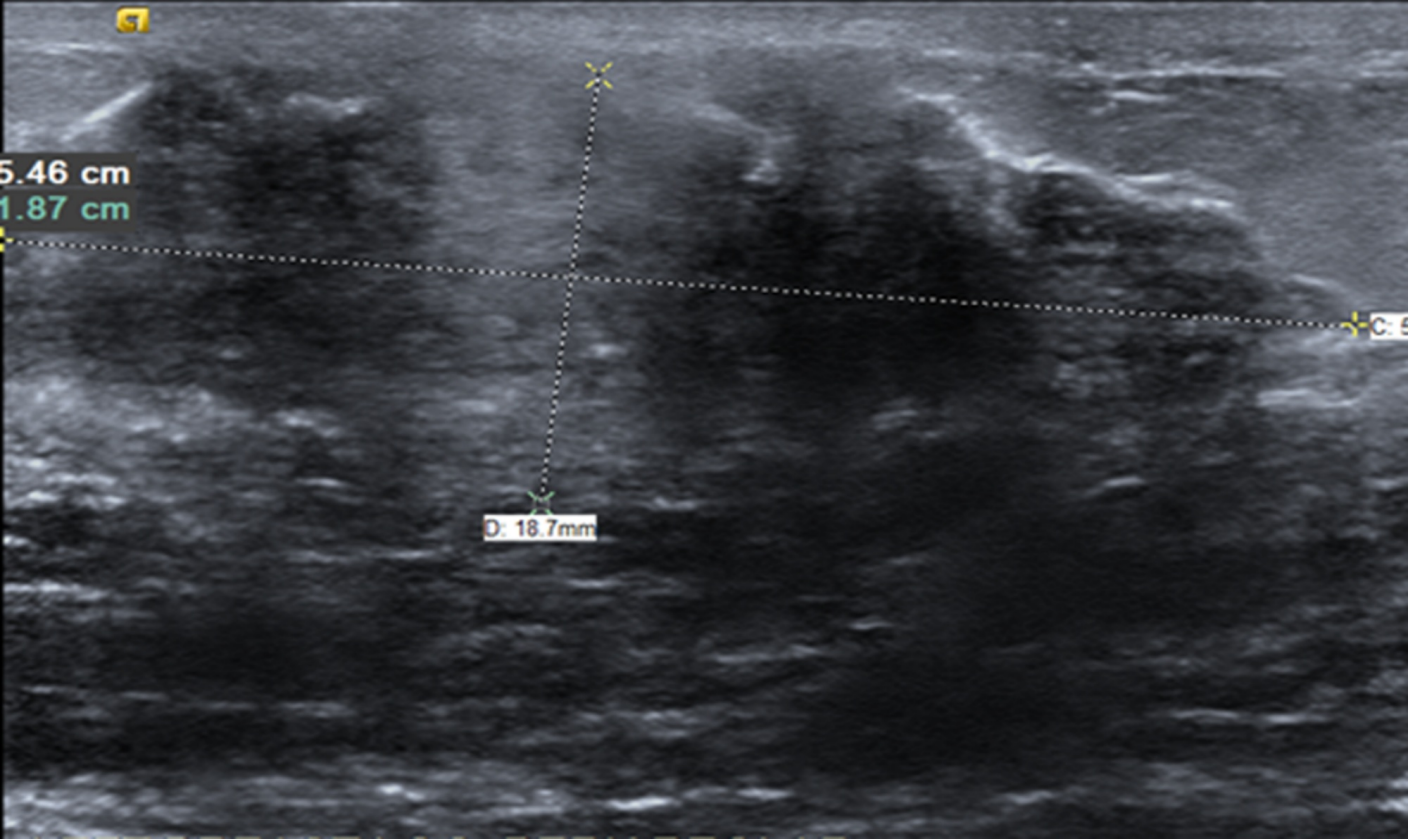
Ultrasound of the left breast Ultrasound of the left breast demonstrating a highly suspicious, ill-defined, deeply hypoechoic area of non-mass-like architectural distortion with acoustic shadowing, measuring 5.4×1.9 cm

We present the case of a 29-year-old, single Saudi female, with an 18-year history of well-controlled, insulin-dependent diabetes mellitus, with no secondary complications. The patient presented to a one-stop breast clinic with a painless, enlarged left breast mass of recent appearance. A breast examination revealed a 4×1 cm, well-circumscribed, retro-areolar hard mass, not fixed and non-tender, with no overlying skin changes. Examination of the right breast and both axilla was unremarkable. Subsequently, the patient was referred to our general surgery service. The patient had a strong family history of breast cancer since her sister was diagnosed at the age of 27, and her mother and aunt were also affected. The affected family members were not tested for BRCA1 and 2 gene mutations. Breast carcinoma, fibroadenoma, and fibrocystic changes of the breast were the initial differential diagnoses considered. The patient’s complete blood count, renal, and liver function tests revealed no abnormalities. Further evaluation with ultrasound (US) imaging revealed a highly suspicious, ill-defined hypoechoic lesion in the left lower outer quadrant consistent with a Breast Imaging, Reporting & Data System (BI-RADS) 4 category (Figure 1).

**Figure 2:**
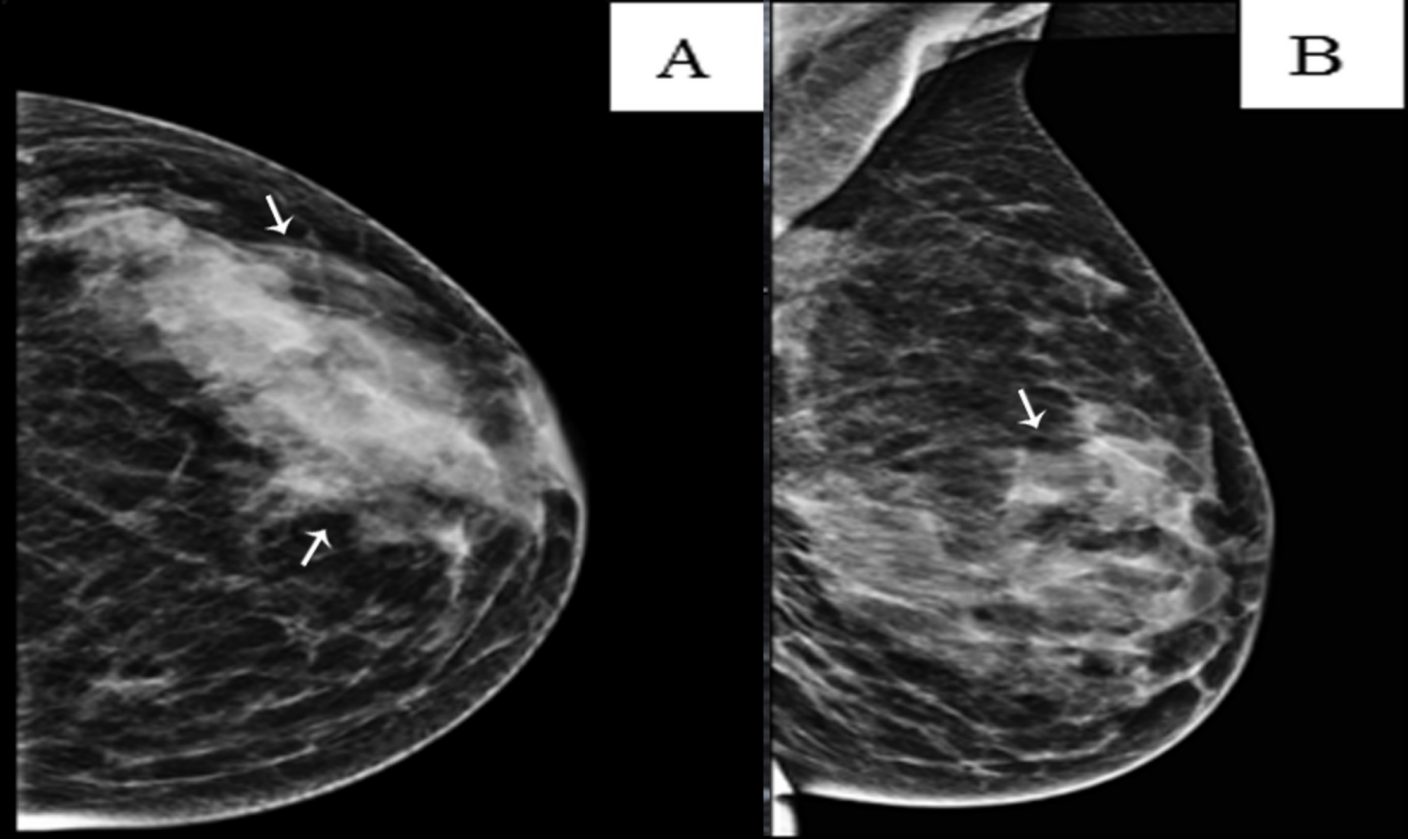
Left breast mammogram Left breast mammogram (A) craniocaudal and (B) mediolateral oblique views showing the typical radiologic appearance of diabetic mastopathy, including dense parenchyma, glandular asymmetry, and no identifiable discrete lesions

Mammographic examination revealed both breasts were of normal shape with bilateral dense glandular parenchyma (breast composition category C) (Figure 2).

**Figure 3:**
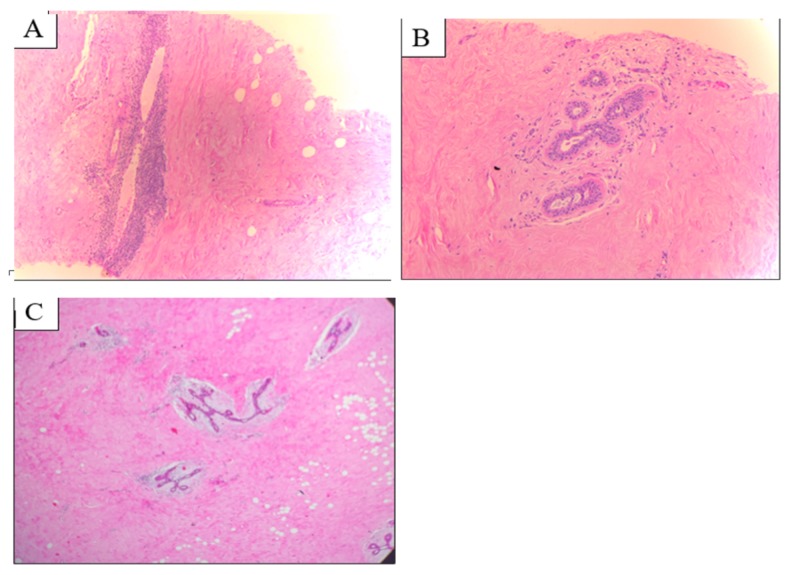
Microscopic features of the DMP lesion (A) and (B): Microscopic examination of the histopathologic specimen (tru-cut needle biopsy), showing dense collagenous stroma and lymphocytic aggregate surrounding ducts and vessels; (C): Section of the excisional biopsy specimen showing extensive fibrosis DMP: diabetic mastopathy

A US-guided, tru-cut biopsy was recommended. Microscopic examination revealed acellular fibrous-sclerotic tissues with normal-looking lobules and ducts, surrounded by dense lymphocytic infiltrate. No evidence of any malignancy was detected (Figures 3A-3B). The pathologist provided an impression of sclerosing lymphocytic mastitis vs. autoimmune mastitis. Subsequently, a diagnosis of diabetic mastopathy was established. Results were discussed with the patient, and an agreement was reached to proceed with an excisional biopsy for further reassurance and exclusion of malignancy. Local surgical excision of the lesion was performed in an uneventful procedure, and the patient experienced smooth postoperative recovery. The histopathological examination revealed extensive fibrosis of the specimen with no cellular atypia (Figure 3C). 

The patient was seen in the clinic two weeks after the surgery and was doing well. Subsequently, the patient was given a six-month follow-up appointment with a US examination. To date, our patient remains well, and no new complaints have been reported.

## Discussion

Our patient had a classical presentation similar to most of the previously reported cases of DMP, with the exception of the family history of breast cancer, since previous studies and reports have not mentioned a history of breast cancer associated with DMP [1-4,6]. According to a systematic literature review published in 2017, there were only approximately 250 cases of diabetic mastopathy reported around the world [6]. This condition represents 1% of all breast pathologies and affects approximately 0.6% to 13% of women with DM I [1,7]. Although DMP has a strong female predilection, a few cases have been reported in males [6,8]. Diabetic mastopathy has most often been described in association with DM I [9]. However, a very similar histological pattern has also been found in patients with thyroid disease, Sjogren's syndrome, and secondary amenorrhea. In those cases, the inflammatory condition was termed lymphocytic mastitis [1,3,9-12]. Moreover, it has also been described in several patients with DM II [13-14]. Patients may be asymptomatic or may present clinically with painless, firm to hard, ill-defined masses. The masses may be solitary or multiple, unilateral or bilateral [2,10,13,15]. Painful lesions were reported in a few cases, and mastectomy was warranted in some [6]. The frequently reported mean patient age at presentation is approximately 45 years, with an average 18-year history of diabetes [6,7]. The diagnosis of diabetic mastopathy is challenging due to its low prevalence and its similar presentation to breast carcinoma [2,4,6-8,12,13,15]. Commonly reported sonographic features include hypoechoic lesions with irregular borders and significant posterior acoustic shadowing. Mammographic examination may reveal non-specific and non-diagnostic findings such as dense parenchyma and asymmetric densities [1,6-7,12-13]. Fine needle aspiration (FNA) typically provides little information. Consequently, the use of a tru-cut biopsy is necessary and provides a definitive histopathological diagnosis. Reports on the paucity of cellular material in FNA has been noted in the medical literature and has become an indicator for the diagnosis [6-7]. Distinctive microscopic pathologic features associated with this condition include dense lymphocytic infiltrates consisting predominantly of B-cells surrounding ducts, lobules, and vessels, dense collagenous stroma (keloid-like), the proliferation of fibroblasts, lobular atrophy, and decreased cellular material [2,6,12-14,16]. These characteristics are concordant with our patient's breast lesion histopathology. Few authors have formulated certain diagnostic criteria for DMP based on reported cases and retrospective studies in recent years [7]. Table 1 shows the selected criteria.

**Table 1:** Proposed criteria for the diagnosis of diabetic mastopathy

Author	Criteria
Camuto, 2000	1. Patient in the premenopausal period with a long history of diabetes mellitus I, with other microvascular complications, with or without an associated history of other auto-immune or endocrine disorders. 2. A presentation of a palpable, non-tender, hard mass which is clinically suspicious for breast carcinoma. 3. Failure of identification of a solid mass or discrete cystic lesion on imaging modalities (ultrasonography-mammogram), and mammogram showing dense parenchyma. 4. Core or excisional biopsy showing dense collagenous stroma (keloid-like), fibrosis, lymphocytic lobulitis, ductitis, and lymphocytic vasculitis.

Although DMP and malignancies may coexist at the same time, no causal relationship has been proved, and most reports describe it as a benign condition [2-4,12,16]. In their study, Valdez et al. reported that B-cells found in DMP disease are not clonal, thus excluding a potential malignant transformation into lymphoma, a previous concern and feared outcome [16]. Another study following patients with DMP for almost 10 years demonstrated no increased risk of breast malignancy [4]. However, studies with longer follow-up may still be required.

Due to DMP's favorable prognosis, the majority of practitioners recommend a non-surgical management approach [6].

Once a histological diagnosis is confirmed, conservative non-surgical management and follow-up are generally accepted and recommended. Although in some cases, it may be needed, surgical excision may lead to a recurrence and should be evaluated on a case-by-case basis [6-7,17-18]. In our case, given the significant family history, the patient opted for surgical management to ensure no malignancy existed. Some researchers have argued that if a DMP mass is not excised, the condition may hinder the detection of a future carcinoma developing within the fibrous region [6]. This is may represent a challenging situation, especially in young patients with a high risk of breast cancer similar to our patient. Therefore, well-informed consent and shared decision-making is important. There has been one previous report of a 48-year-old woman with breast carcinoma within a previously diagnosed DMP mass. The patient was followed conservatively after a histological diagnosis of DMP, noted an increase in the size of the mass, and subsequently returned for revaluation [19]. A diagnosis of infiltrating ductal carcinoma was made after a wide surgical excision [19]. Patient education regarding self-breast exams is highly important when conservative management is implemented, and it should help decrease the risk of missing a cancerous mass if it develops [18-19].

## Conclusions

Diabetic mastopathy is less prevalent than breast malignancy. However, it should be kept in mind when caring for all patients with long-standing DM who present with a breast lump. If diagnosed, the management approach must take into account its benign nature, thus alleviating patient anxiety and avoiding unnecessary surgical intervention.
